# Network analysis of psychological factors related to academic pressure faced by medical students in the central and highland regions of Vietnam

**DOI:** 10.1080/10872981.2021.2007577

**Published:** 2021-11-25

**Authors:** Minh Tu Nguyen, Thanh Gia Nguyen, Tho Thi Anh Tran, Thi Thanh Nhan Nguyen, Dinh Duong Le, Thi Thanh Binh Nguyen, Huu Hai Hoang, Thi Linh Dan Ho, Binh Thang Tran

**Affiliations:** a Undergraduate Training Office, University of Medicine and Pharmacy, Hue University, Hue City, Vietnam; b Faculty of Public Health, University of Medicine and Pharmacy, Hue University, Hue City, Vietnam; c Department of Gastroenterology and Hepatology, Nghe An Oncology Hospital, Nghe An, Vietnam; d Department of Pediatrics, University of Medicine and Pharmacy, Hue University, Hue City, Vietnam; e Student’s Affairs Office, University of Medicine and Pharmacy, Hue University, Hue City, Vietnam

**Keywords:** Medical training, psychological beliefs, attitudes, vietnamese students, network analysis

## Abstract

Medical students experience extensive pressure during their undergraduate courses. Given the complex associations between psychological factors in association with academic pressure. We investigated the study with objectives: To examine psychological factors related to academic pressure by analysing interactions between ‘study motivation’, ‘study environment’, ‘study conditions’, ‘teacher quality’, ‘training programme’, ‘management system’, ‘evaluation’, and ‘extracurricular activities’ using a network analysis approach. A total of 878 medical students majoring in general medicine from the first, third, and fifth years of a six-year course at the largest medical university in central and highland regions of Vietnam were involved in this cross-sectional study. The approach used was convenient cluster sampling with a self-administered questionnaire by the participants. Network analysis for pairwise correlations between psychological factors was estimated . Important factors in the network analysis were calculated using centrality indices including node strength (S), closeness (C), and betweenness (B). The higher score of S, C, and B indicate the more importance of the node. The results obtained from the network analysis of eight psychological factors showed that ‘teaching quality’ was mostly connected with other factors overall, while the ‘training programme’ was seen in both genders and freshman students. ‘Study conditions’ and ‘training programme’ were mostly connected with other factors in junior and senior students, respectively. The strong pairwise correlation was confirmed: management system and evaluation activity, followed by study environment and study conditions, and teaching quality and training programme. Additionally, nodes with high centrality were shown to be ‘management system’ (S = 0.97, C = 0.019, B = 1), and ‘training programme’ (S = 0.96, C = 0.021, B = 4). Our study findings indicate that satisfaction with the training programme amongst eight psychological factors is the most important factor affecting academic pressure among medical students. The training programme is linked with teaching quality, whereas the management system is correlated with evaluation activity.

## Introduction

Due to isolation from their family, the university stage can be a stressful period for adolescents, with unhealthy behaviours, such as physical inactivity and poor quality diet, or physiological and psychological changes at both the social and educational levels [1,2]. It is estimated that approximately 80% of university students worldwide experience stress after examinations or submitting papers and assignments [3]. Among these stresses, academic pressure is prevalent, particularly in medical students, due to the high expectations of the individuals, not least on their parents, and the demands of university life [3]. Several scales have been developed to measure academic pressure among adolescents and students [4]; however, levels of academic pressure may differ between various streams, such as humanities, commerce, management, or science [5]. It has been reported that academic stress among Indian students using the Academic Stress Scale was observed to be statistically significantly higher in students in the science stream than in the commerce stream and also in the management and commerce streams than in the humanities stream [5]. Unlike other majors, medical students may experience a greater level of pressure during their undergraduate course than same-age peers, despite similar or healthier profiles [6–9]. For example, the prevalence of stress among medical students was found to be higher than in the general population, with an estimated 31.2% in three British universities, 61.4% in a Thailand school, and 41.9% in a Malaysian school [10].

Furthermore, many previous studies indicate that academic stress is associated with psychological beliefs and attitudes factors such as study motivation [11], study environment, study conditions [12], teacher quality [13], training programme [14,15], management system [16], evaluation, and extracurricular activities [17]. These factors may independently and/or together significantly impact the level of academic stress. Further analysis is needed to assess the interrelationships between these factors and understand the academic pressure faced by students. Such an analysis would provide evidence for stakeholders, enabling a prioritised intervention strategy to be put in place and aligned with available resources aimed at minimising/eliminating the impact of academic stress.

Psychological symptoms potentially stemming from academic pressure have been reported in some universities in Vietnam; for example, the prevalence of depression amongst students is higher than in the general Vietnamese population [18,19]. Until now, there has been no reported study using a network analysis approach to assess the interrelationships between these factors.

In psychological sciences research, network analysis has gained substantial attention for its ability to provide a picture of the interrelationships between factors [20–23]. In general, the network structure consists of nodes representing individual variables of interest, with edges representing the correlation between them [20–23]. Given the complex associations between psychological factors, we carried out this study to examine the interactions between study motivation, study environment and study conditions, teacher quality, training programme, management system, and evaluation and extracurricular activities in a network analysis approach. Knowledge of these interactions will help in providing appropriate strategies to improve the students’ psychological beliefs and attitudes and to reduce the level of academic pressure experienced by medical students in Vietnam.

## Study methods

### Study participants

Taking account of previous findings on levels of exposure to academic stress and mental health issues, we carried out a cross-sectional study with general practice majors in the first, third and fifth academic years (corresponding to freshman, juniors, and seniors) exposed to different learning experiences at medical university in their respective curriculum streams. It is recognised that the first year is when students are simply trying to adapt and gain a large amount of knowledge; the third year is when students transfer from basic to pre-clinical subjects, with initial exposure to clinical visits; and the fifth year is when students become more independent in their clinical knowledge and skills. This study was conducted from October to November 2020 at the University of Medicine and Pharmacy, Hue University (Hue UMP), Hue City, Vietnam. Hue UMP is the largest public medical university in the central and highlands regions of Vietnam, with various majors, including a six-year programme for general medicine. The university is also responsible for training in human resources for the regions of the central and highlands of Vietnam. Details of the curriculum for medical training are well-documented, with the first three years in basic science and the last three years in clinical practice [24].

### Sample and data collection

Given in fact that the sample size for the network model range up to 350, which is properly observed high specificity, moderate sensitivity, and edge weights correlations [25]. Therefore, some 878 students out of 1171 students in total (1^st –^ freshman, 3^rd –^ junior, 5^th^- senior year) in the 2020–2021 academic year were invited to participate in this survey. For convenience, we first selected all classes in each year and then selected over 75% of the students from each year to give a representative population. Data were collected via a self-administered questionnaire with support from researchers. Researchers explained details about the study and guided students in how to fill out the questionnaire. Students then put their completed surveys into the collection box. The survey took 10–15 minutes to complete.

### Measurements

The survey consisted of questions on (1) demographic characteristics, (2) lifestyle behaviours, and (3) psychological beliefs and attitudes.

Demographic characteristics included: age information (years); gender (male, female); academic stage (freshman, junior, senior); type of accommodation (living with family, alone, with a roommate, with relatives, and other); monthly support from family (<$67, $67 to <$111, $111 to <$156, ≥$156, and other); and academic grading (excellent, very good, good, fairly good, average, weak, and poor).

Lifestyle behaviours included: daily sleeping time (hours); tobacco smoking (yes/no); alcohol consumption (yes/no); and physical activity (never, 1–2 times/month, 3–4 times/month, 1–2 times/week, and ≥3 times/week).

Psychological beliefs and attitudes included: 53 questions regarding study motivation (5 questions); study environment (8 questions); study conditions (7 questions); teacher quality (9 questions); training programme (7 questions); management system (6 questions); evaluation activity (5 questions); and extracurricular activity (6 questions) [26,27]. Student responses were obtained using a 5-point Likert scale: (1) strongly disagree, (2) disagree, (3) neither agree nor disagree, (4) agree, and (5) strongly agree [26–28]. Higher scores indicate a greater degree of satisfaction. Details of the questions are provided in the **Supplementary Appendix**.

### Statistical analysis

Descriptive statistics were calculated as mean and standard deviations for continuous variables, and as counts and percentages for categorical variables. The normal distribution of psychological factor scores was assessed using a Shapiro-Wilk test. Given that females tend to experience greater mental troubles than males, all analyses were conducted for the total study population and subgroups of males and females [29]. Moreover, students from different academic stages may also experience stress differently, and thus we performed subgroup analyses by gender and academic stage. Differences between males and females as well as academic stage were assessed using a t-test, analysis of variance (ANOVA), and chi-square test.

In this study, the network analysis for the interrelationships between psychological factors was performed using a Gaussian graphical model (GGM). Given that the scores of most of the psychological factors were not normally distributed (**Table S1**, *p* < 0.05), the scores of eight psychological factors were first log-transformed and then standardised to improve the normal distribution. We implemented the regularization in the ‘qgraph’ package, in which pairwise correlations for the network structure were estimated using the extended Bayesian information criterion (EBIC) set at 0.5, and the Glasso algorithm [23,30]. The EBIC identified Glasso tuning parameters for the network of psychological factors among total, male, female, freshman, junior, and senior students, with values of 0.061, 0.067, 0.060, 0.069, 0.053, and 0.066, respectively.

To measure the importance of factors included in the network, we calculated centrality indices including node strength (S, the absolute sum of edge weights connected to a node); closeness (C, the average distance from the node to all other nodes in the network); and betweenness (B, the number of times that a node lies on the shortest path between two other nodes) [23,31]. Furthermore, we examined the stability of node centralities by bootstrapping 80% of the original sample with a replacement. This process was implemented in the ‘bootnet’ package, stability was quantified through a CS coefficient [32]. By default, the CS coefficient identified the maximum proportions to retain a correlation of 0.7 in at least 95% of the sample [23]. In addition, the accuracy and certainty of all results in the networks also were evaluated to provide a sufficient conclusion by examining the bootstrapped different tests for edge-weight and centrality indices [23]. All the statistical analyses were implemented in R version 3.6.0 (R Foundation for Statistical Computing, Vienna, Austria).

### Ethical approval

The research proposal and tools were approved by the Ethics Committee for Biomedical Researches of University of Medicine and Pharmacy, Hue Universiy (No H2020/443, dated 30 August 2020). The students who agreed to participate signed informed consent forms and were given an information sheet. They could refuse to participate in the survey at any time.

## Results

The general characteristics and psychological factor scores of 878 medical students are summarised in Table 1. The sample participants were equally distributed between freshman (first year), junior (third year), and senior students (fifth year). The mean age of the students was 21.3 years (± 1.9). Most participants live alone (64.7%), receive monthly support from their family of $67-$156 (72.9%), have good or very good academic grading (79.5%) and do not smoke tobacco (97.6%) or drink alcohol (78.0%). The mean/total scores of psychological beliefs and attitudes, respectively, were 18.6/25, 24.4/40, 21.7/35, 31.5/45, 22.7/35, 19.0/30, 17.0/25, and 18.3/30 for study motivation, study environment, study conditions, teacher quality, training programme, management system, evaluation activity, and extracurricular activity. There were statistically significant differences between male and female students in type of accommodation, monthly support from family, smoking, alcohol consumption, and physical activity (*p* ≤ 0.001). Male students were more likely to live with family or relatives, receive higher support from family, smoke, drink alcohol, and regularly perform physical activity. Freshman, junior, and senior students were equally distributed, corresponding to approximately 60% of the total students in each year.

**Table 1.** General characteristics and psychological factor scores of study participants

Variable	Total (N = 878)	Male (N = 376)	Female (N = 502)	*p*-value a	Freshman (N = 255)	Junior (N = 297)	Senior (N = 326)	*p*-value b
Age (years) (Mean ± SD)	21.3 ± 1.9	21.2 ± 2.0	21.4 ± 1.9	0.13	19.0 ± 0.7	21.2 ± 0.9	23.3 ± 0.7	<0.001
Gender								
Male	376 (42.8%)	376 (100%)	0 (0.0%)	NA	124 (48.6%)	119 (40.1%)	133 (40.8%)	0.08
Female	502 (57.2%)	0 (0.0%)	502 (100%)		131 (51.4%)	178 (59.9%)	193 (59.2%)	
Academic standing								
Freshman (First)	255 (29.0%)	124 (33.0%)	131 (26.1%)	0.08	255 (100%)	0 (0.0%)	0 (0.0%)	NA
Junior (Third)	297 (33.8%)	119 (31.6%)	178 (35.5%)		0 (0.0%)	297 (100%)	0 (0.0%)	
Senior (Fifth)	326 (37.1%)	133 (35.4%)	193 (38.4%)		0 (0.0%)	0 (0.0%)	326 (100%)	
Type of housemate								
With family	97 (11.0%)	59 (15.7%)	38 (7.6%)	<0.001	31 (12.2%)	31 (10.4%)	35 (10.7%)	<0.001
Alone	568 (64.7%)	237 (63.0%)	331 (65.9%)		141 (55.3%)	202 (68.0%)	225 (69.0%)	
With roomate	194 (22.1%)	67 (17.8%)	127 (25.3%)		74 (29.0%)	60 (20.2%)	60 (18.4%)	
With relatives	13 (1.5%)	10 (2.7%)	3 (0.6%)		9 (3.5%)	3 (1.0%)	1 (0.3%)	
Other	6 (0.7%)	3 (0.8%)	3 (0.6%)		0 (0.0%)	1 (0.3%)	5 (1.5%)	
Monthly support								
<67 USD	84 (9.6%)	47 (12.5%)	37 (7.4%)	0.001	34 (13.3%)	22 (7.4%)	28 (8.6%)	0.05
67-<111 USD	306 (34.9%)	109 (29.0%)	197 (39.2%)		91 (35.7%)	102 (34.3%)	113 (34.7%)	
111-<156 USD	334 (38.0%)	144 (38.3%)	190 (37.8%)		87 (34.1%)	131 (44.1%)	116 (35.6%)	
≥156 USD	151 (17.2%)	73 (19.4%)	78 (15.5%)		42 (16.5%)	42 (14.1%)	67 (20.6%)	
Other	3 (0.3%)	3 (0.8%)	0 (0.0%)		1 (0.4%)	0 (0.0%)	2 (0.6%)	
Academic grading								
Excellent	48 (5.5%)	21 (5.6%)	27 (5.4%)	0.46	14 (5.5%)	25 (8.4%)	9 (2.8%)	<0.001
Very good	271 (30.9%)	116 (30.9%)	155 (30.9%)		71 (27.8%)	127 (42.8%)	73 (22.4%)	
Good	427 (48.6%)	173 (46.0%)	254 (50.6%)		122 (47.8%)	120 (40.4%)	185 (56.7%)	
Fairly good	108 (12.3%)	52 (13.8%)	56 (11.2%)		42 (16.5%)	21 (7.1%)	45 (13.8%)	
Average	22 (2.5%)	13 (3.5%)	9 (1.8%)		5 (2.0%)	4 (1.3%)	13 (4.0%)	
Weak	2 (0.2%)	1 (0.3%)	1 (0.2%)		1 (0.4%)	0 (0.0%)	1 (0.3%)	
Sleeping time (hours)	7.3 ± 1.5	7.4 ± 1.7	7.3 ± 1.3	0.11	7.6 ± 1.5	7.1 ± 1.2	7.4 ± 1.6	0.18
Smoking								
Yes	21 (2.4%)	21 (5.6%)	0 (0.0%)	<0.001	2 (0.8%)	4 (1.3%)	15 (4.6%)	0.004
No	857 (97.6%)	355 (94.4%)	502 (100.0%)		253 (99.2%)	293 (98.7%)	311 (95.4%)	
Alcohol drinking								
Yes	193 (22.0%)	143 (38.0%)	50 (10.0%)	<0.001	42 (16.5%)	71 (23.9%)	80 (24.5%)	0.04
No	685 (78.0%)	233 (62.0%)	452 (90.0%)		213 (83.5%)	226 (76.1%)	246 (75.5%)	
Physical activity								
Never	62 (7.1%)	16 (4.3%)	46 (9.2%)	<0.001	6 (2.4%)	31 (10.4%)	25 (7.7%)	<0.001
1–2 times/months	220 (25.1%)	53 (14.1%)	167 (33.3%)		36 (14.1%)	87 (29.3%)	97 (29.8%)	
3–4 times/months	246 (28.0%)	114 (30.3%)	132 (26.3%)		85 (33.3%)	88 (29.6%)	73 (22.4%)	
1–2 times/week	196 (22.3%)	108 (28.7%)	88 (17.5%)		68 (26.7%)	59 (19.9%)	69 (21.2%)	
≥3 times/week	154 (17.5%)	85 (22.6%)	69 (13.7%)		60 (23.5%)	32 (10.8%)	62 (19.0%)	
Study motivation (Mean ± SD)	18.6 ± 3.1	18.6 ± 3.1	18.6 ± 3.0	0.90	19.3 ± 3.1	18.5 ± 3.0	18.0 ± 3.0	<0.001
Study environment (Mean ± SD)	24.4 ± 4.7	24.5 ± 5.0	24.3 ± 4.4	0.62	26.6 ± 4.3	23.7 ± 4.3	23.3 ± 4.6	<0.001
Study condition (Mean ± SD)	21.7 ± 4.4	21.9 ± 4.7	21.7 ± 4.3	0.48	24.0 ± 4.0	20.8 ± 4.3	20.9 ± 4.3	<0.001
Teacher quality (Mean ± SD)	31.5 ± 4.9	31.4 ± 5.4	31.6 ± 4.4	0.52	32.3 ± 5.0	31.2 ± 4.8	31.1 ± 4.8	0.006
Training program (Mean ± SD)	22.7 ± 4.5	23.0 ± 4.6	22.5 ± 4.3	0.15	24.7 ± 3.9	21.9 ± 4.3	21.9 ± 4.6	<0.001
Managing system (Mean ± SD)	19.0 ± 3.8	19.0 ± 4.2	19.0 ± 3.5	0.82	20.3 ± 3.6	18.6 ± 3.8	18.3 ± 3.9	<0.001
Evaluation activity (Mean ± SD)	17.0 ± 3.0	17.1 ± 3.3	16.8 ± 2.8	0.19	17.4 ± 2.9	17.0 ± 3.0	16.6 ± 3.1	0.002
Extracurricular activity (Mean ± SD)	18.3 ± 4.0	18.3 ± 4.3	18.4 ± 3.7	0.77	19.9 ± 3.4	17.9 ± 3.8	17.4 ± 4.1	<0.001

^a^*p*-values are calculated from a t-test for continuous variables and a chi-square test for categorical variables for the differences between males and females. ^b^*p*-values are calculated from the ANOVA test for continuous variables and a chi-square test for categorical variables for the differences between academic standings. Bold fonts indicate statistical significance. NA, not applicable.

**Figure 1.**
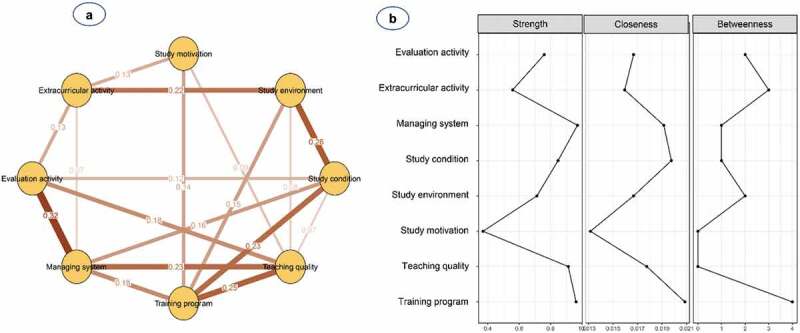
**(a)** Network structure and **(b)** centrality indices of psychological beliefs and attitudes among a total of 878 medical students

Figure 1a and **Table S2** show the network structure of eight psychological beliefs and attitudes among study participants. The pairwise correlation was observed to be strongest for the management system and evaluation activity (0.32), followed by study environment and study conditions (0.26), and teaching quality and training programme (0.25). Teaching quality was mostly connected with other factors, except for extracurricular activity, while study motivation was the most peripheral and least connected node, and only linked to teaching quality (0.09) and extracurricular activity (0.13). Additionally, nodes with high centrality were shown to be management systems (S = 0.97, C = 0.019, B = 1) and training programmes (S = 0.96, C = 0.021, B = 4) (Table 2 and Figure 1b).

**Table 2.** Centrality indices of the multidimensional network of psychological factors in a sample of 878 medical students

Population	Factor	Strength	Closeness	Betweenness
Total	Study motivation	0.37	0.013	0
	Study environment	0.71	0.017	2
	Study condition	0.85	0.02	1
	Teaching quality	0.91	0.018	0
	Training program	0.96	0.021	4
	Managing system	0.97	0.019	1
	Evaluation activity	0.76	0.017	2
	Extracurricular activity	0.56	0.016	3
Male	Study motivation	0.28	0.013	0
	Study environment	0.83	0.018	2
	Study condition	0.74	0.016	0
	Teaching quality	0.87	0.017	0
	Training program	1.00	0.022	4
	Managing system	1.04	0.02	3
	Evaluation activity	0.79	0.017	2
	Extracurricular activity	0.52	0.017	2
Female	Study motivation	0.42	0.014	0
	Study environment	0.76	0.019	3
	Study condition	0.90	0.022	6
	Teaching quality	0.75	0.018	2
	Training program	0.90	0.019	3
	Managing system	0.85	0.019	0
	Evaluation activity	0.72	0.016	2
	Extracurricular activity	0.34	0.015	0
Freshman	Study motivation	0.135	0.012	0
	Study environment	0.591	0.016	0
	Study condition	0.735	0.020	2
	Teaching quality	1.241	0.026	11
	Training program	0.957	0.020	4
	Managing system	0.896	0.020	1
	Evaluation activity	0.844	0.022	1
	Extracurricular activity	0.314	0.013	0
Junior	Study motivation	0.206	0.011	0
	Study environment	0.714	0.022	10
	Study condition	0.992	0.023	10
	Teaching quality	0.700	0.019	0
	Training program	0.773	0.019	1
	Managing system	0.890	0.017	1
	Evaluation activity	0.522	0.017	0
	Extracurricular activity	0.474	0.016	6
Senior	Study motivation	0.203	0.013	0
	Study environment	0.733	0.020	5
	Study condition	0.744	0.016	0
	Teaching quality	0.854	0.022	0
	Training program	1.000	0.021	8
	Managing system	0.917	0.022	3
	Evaluation activity	0.982	0.019	2
	Extracurricular activity	0.376	0.015	0

Nodes reflect psychological factors, and edges reflect the conditional dependencies between psychological factors. The thickness of edges represents the strength of correlations.

**Figure 2.**
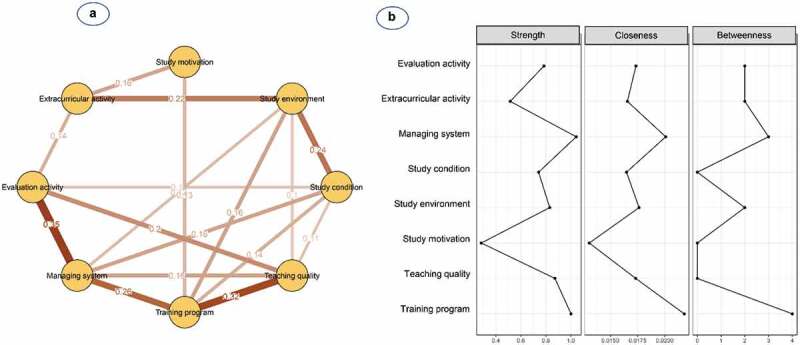
**(a)** Network structure and **(b)** centrality indices of psychological beliefs and attitudes among 376 medical male students

Figure 2a and **Table S2** show the network structure of eight psychological beliefs and attitudes in male students. The pairwise correlation was observed to be strongest for the management system and evaluation activity (0.35), followed by teaching quality and training programme (0.32), and training programme and management system (0.26). The training programme was one of the most connected nodes, whereas study motivation was the most peripheral node and was only linked to the training programme (0.13) and extracurricular activity (0.16). Additionally, nodes with high centrality were shown to be management systems (S = 1.04, C = 0.020, B = 3) and training programmes (S = 1.00, C = 0.022, B = 4) (Table 2 and Figure 2b).

Nodes reflect psychological factors, and edges reflect the conditional dependencies between psychological factors. The thickness of edges represents the strength of correlations.

**Figure 3.**
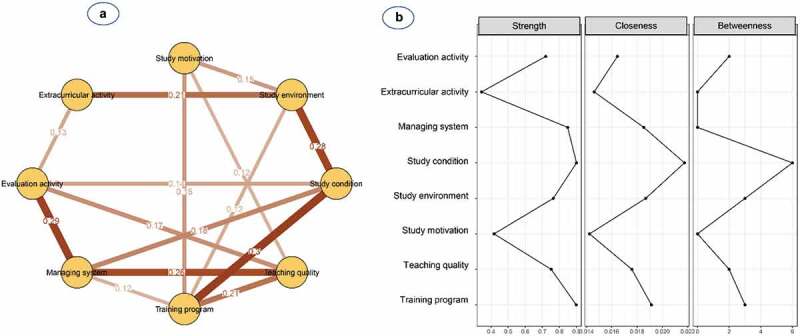
**(a)** Network structure and **(b)** centrality indices of psychological beliefs and attitudes among 502 medical female students

Figure 3a and **Table S2** show the network structure of eight psychological beliefs and attitudes in female students. The pairwise correlation was observed to be strongest for study conditions and training programme (0.30), followed by the management system and evaluation activity (0.29), and study environment and study conditions (0.28). The training programme was the most connected node, whereas extracurricular activity was the most peripheral node and only linked to the study environment (0.21) and evaluation activity (0.13). Additionally, nodes with high centrality were shown to be study conditions (S = 0.90, C = 0.019, B = 3) and training programme (S = 0.90, C = 0.019, B = 3) (Table 2 and Figure 3b).

Nodes reflect psychological factors, and edges reflect the conditional dependencies between psychological factors. The thickness of edges represents the strength of correlations.

**Figure 4.**
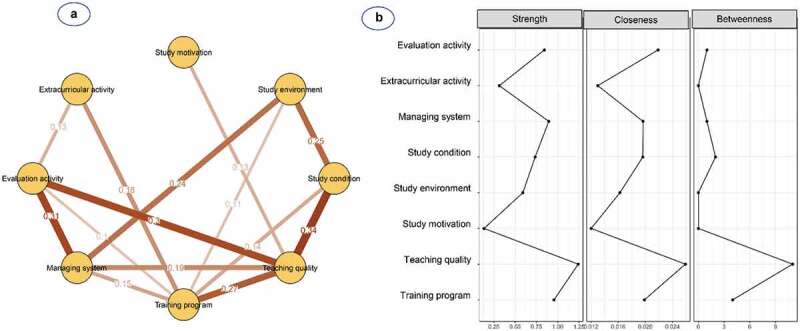
**(a)** Network structure and **(b)** centrality indices of psychological beliefs and attitudes among 255 freshmen medical students

Figure 4a and **Table S2** show the network structure of eight psychological beliefs and attitudes in freshmen. The pairwise correlation was observed to be strongest for study conditions and teaching quality (0.34), followed by teaching quality and training programme (0.27) and study environment and study conditions (0.24). The training programme was the most connected node, whereas study motivation was the most peripheral node and only linked to teaching quality (0.13). Additionally, nodes with high centrality were shown to be teaching quality (S = 1.24, C = 0.026, B = 11) and evaluation activity (S = 0.84, C = 0.022, B = 1) (Table 2 and Figure 4b).

Nodes reflect psychological factors, and edges reflect the conditional dependencies between psychological factors. The thickness of edges represents the strength of correlations.

**Figure 5.**
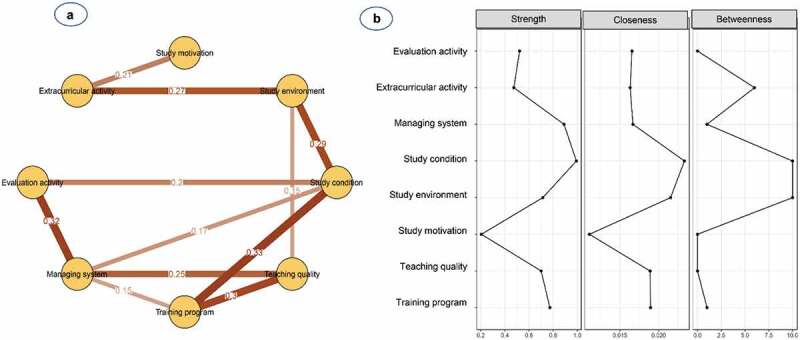
**(a)** Network structure and **(b)** centrality indices of psychological beliefs and attitudes among 297 junior medical male students

Figure 5a and **Table S2** show the network structure of eight psychological beliefs and attitudes in junior students. The pairwise correlation was observed to be strongest for study conditions and training programme (0.33), followed by the management system and evaluation activity (0.32) and teaching quality and training programme (0.30). Study conditions were the most connected node, whereas study motivation was the most peripheral node and only linked to extracurricular activity (0.21). Additionally, nodes with high centrality were shown to be study conditions (S = 0.99, C = 0.023, B = 10) and study environment (S = 0.71, C = 0.022, B = 10) (Table 2 and Figure 5b).

Nodes reflect psychological factors, and edges reflect the conditional dependencies between psychological factors. The thickness of edges represents the strength of correlations.

**Figure 6.**
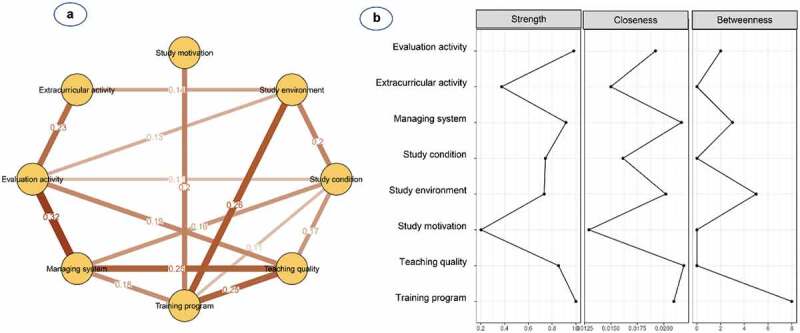
**(a)** Network structure and **(b)** centrality indices of psychological beliefs and attitudes among 326 senior medical female students

Figure 6a and **Table S2** show the network structure of eight psychological beliefs and attitudes in senior students. The pairwise correlation was observed to be strongest for the management system and evaluation activity (0.32), followed by study environment and training programme (0.26), teaching quality and training programme (0.25), and teaching quality and management system (0.25). Study conditions and training programmes were the most connected nodes, whereas study motivation was the most peripheral node and only linked to the training programme (0.20). Additionally, nodes with high centrality were shown to be training programmes (S = 1.00, C = 0.021, B = 8), and management systems (S = 0.92, C = 0.022, B = 3) (Table 2 and Figure 6b).

Nodes reflect psychological factors, and edges reflect the conditional dependencies between psychological factors. The thickness of edges represents the strength of correlations.

**Figure 7.**
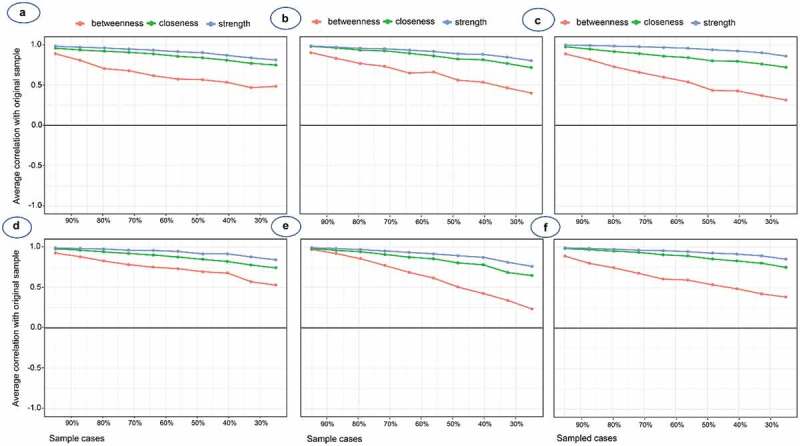
Graphical depiction for the stability of network centrality indices of **(a)** total, **(b)** male, **(c)** female, **(d)** freshmen, **(e)** junior; and **(f)** senior students

Furthermore, Table 3 presents the CS coefficients for the maximum proportions to retain a correlation of 0.7 between node centrality and the original sample. As a result, node strength appeared to be more stable than node closeness and betweenness, regardless of total study participants and subgroups of gender and academic standing (Figure 7). The accuracy of pairwise correlation and node centrality was also investigated (Figure S1-S6). Pairwise correlations amongst eight factors have confirmed the pattern with highly consistent between sampled data and bootstrap mean. With a different portion of sample size in the simulation, average correlations between centrality indices of networks sampled with subjects dropped and the original sample were robustly consistent.

**Table 3.** Maximum proportions to retain correlation of 0.7 between node centrality and the original sample

Population	Strength	Closeness	Betweenness
Total	74.9%	43.8%	5.0%
Male	67.3%	36.3%	0
Female	51.6%	28.3%	5.0%
Freshman	59.6%	43.9%	5.1%
Junior	51.5%	28.3%	12.8%
Senior	59.5%	36.2%	4.9%

The percentage of the original data sampled is on the x-axis and the correlation of node centrality with the original sample is on the y-axis. Higher correlations indicate more stable indices.

## Discussion

This study was undertaken among 878 surveyed general medicine students to investigate the intercorrelations between psychological factors related to academic pressure. Overall, our study indicates that teaching quality is the most prominent linkage with other psychological factors. The training programme was found to be the most connected with other factors in both males and females and was also seen in freshman students, while study conditions, and study conditions and training programmes were commonly seen in junior and senior students, respectively. The highest correlation was found between two psychological components in the network, including management system and training programme in all participants, in particular also in males and senior students; study conditions and training programme in females; teaching quality and evaluation activity in freshmen, and study conditions and study environment in juniors.

Many previous studies indicate that training programmes, teaching quality, and evaluation activity is significantly associated with student satisfaction. For example, assuming that the components of a training programme, including course design, course content support, course assessment, and instructor characteristics are well-planned – in this case, student satisfaction could be significantly increased with a learning system [33]. Using new approaches for teaching training programmes, such as flipped classroom teaching and learning activities, indicates a positive effect on student academic performance [34]. Applied problem-based learning methodology in which instructors are responsible for creating learning opportunities that facilitate and enhance student skills in deep learning, also increase student satisfaction [35]. On the other hand, study condition was an important factor influencing student satisfaction. Weerasinghe et al. (2018) reported that the quality of lecture room facilities, library facilities, accommodation facilities, employment facilities, and entertainment facilities in universities strongly affects the degree of student satisfaction [36]. If well-planned, all these factors taken together possibly lead to the minimization of psychological-related academic problems in students. However, the assessment of the interrelationships between these factors is extremely limited. Our findings indicate that satisfaction with the purpose and workload of the training programme was the most central factor. Furthermore, the training programme was found to be strongly correlated to satisfaction with instructors’ knowledge and skills; all considered factors of academic knowledge. Moreover, there was a strong correlation of school-related factors, such as satisfaction of exam evaluation, support from the department, and tuition policy; all belonging to the management system and evaluation activity. Still required is an overall evaluation of the programme that considers minimization/elimination of potential factors impacting student academic stress but still ensures that the expected learning outcomes of the programme are delivered.

In this study, the scores of psychological beliefs and attitudes were not significantly different between male and female students. In the subgroup analysis by gender, the training programme is the most central node. However, in males, training programme satisfaction was more strongly correlated with teaching quality than with study conditions; whereas, in females, training programme satisfaction was more strongly correlated with study conditions than with teaching quality. In contrast, a strong correlation between the management system and evaluation activity was observed in both males and females. Furthermore, study motivation and extracurricular activity were the most peripheral nodes in the network structure of both the total study population and gender-specific population. A previous study reported higher overall satisfaction with a training programme by females than by males [37]. Moreover, females generally experience higher levels of depression, frustration, and anxiety, leading to higher levels of stress than males [38,39], which could be explained by factors such as physiological differences in genetic vulnerability or hormones, sensitivity to events, and self-concepts of masculinity and femininity [29]. Further studies are needed to understand this issue more deeply.

Interestingly, we found central node of psychological factors that affect the academic pressure were different among students in the first, third, and fifth years. The training programme, study conditions, and study conditions and training programme were central notes of the factors for 1st, 3rd, and 5th-year students, respectively. These could be explained based on their experiences on the course. For 1st students, the training programme was an important factor. They entered the medical course after passing a strict national examination. The changing of environmental study and increasing academic pressure from the training programme were caused depression in medical students [40,41]. Factors that were strongly correlated with each other could be influenced by the high school environment where they studied. Regarding 3^rd^ year students, study conditions and environments (e.g., spacious study and practice rooms, materials, and curriculum of each subject) were important factors because they have started learning and receiving their first clinical experiences from this academic year. Regarding 5th year students, study conditions and training programmes were the main factors due to their demand for learning and receiving more clinical experiences [18]. For every school year, therefore, it is necessary to identify factors associated with academic pressure among medical students of every class year of the course to prevent potential psychological problems.

Although several factors associated with psychological distress have been identified, mostly focused on demographics and lifestyle such as low financial support, sleeping quality, eating behaviour, and physical inactivity – the factors of beliefs and attitudes have not been adequately examined in a quantitative approach [42–49]. According to the authors’ knowledge, this is the first study to apply network analysis to examine the complex intercorrelations between psychological factors in medical students in Vietnam. Network analysis in a GGM approach removed the indirect effects by calculating the pairwise correlations between two factors in the independence of the remaining factors in the network [50–52]. Additionally, we obtained information on each psychological belief and attitude via several questions to improve the evaluation of each factor as it relates to academic pressure. Furthermore, an equal distribution of the study participants among freshman, junior, and senior students supported our representative findings in medical students of different academic standing.

Despite the strengths evidenced, some limitations of the study need to be considered. First, although our questionnaire was reviewed carefully to most closely reflect the current study environment in Vietnam, we acknowledge that a lack of validation might raise questions about the adequacy of all factors related to academic pressure and therefore, potentially affect the validity of our results. Second, the cross-sectional study design only observed the associations between factors rather than their causal relationship in a directed acyclic graph performance. Third, this study only included students from a medical university in the central region of Vietnam, which may not be representative of the psychological beliefs and attitudes among medical students from universities in other cities and provinces with different lifestyles, behaviours, and cultures.

This finding, though preliminary, suggests that the schools should regularly review and strengthen, particularly in focusing on the pairs factors such as management system and evaluation in general, as well as the present study condition and teaching quality, study condition and training program in the junior students, study environment and training program in the senior students. These improvements are beneficial for students’ academic performance and well-being. In summary, we found that satisfaction with the training programme and with study conditions were the most central nodes of psychological factors affecting academic pressure among medical students in Vietnam. The training programme was strongly correlated with teaching quality, whereas the management system was strongly correlated with evaluation activity. Further large-scale studies are needed to confirm the findings of the study.

## Supplementary Material

Supplemental Material

## References

[1] Chacon-Cuberos R, Zurita-Ortega F, Olmedo-Moreno EM, et al. Relationship between academic stress, physical activity and diet in university students of education. Behavioral Sciences (Basel, Switzerland). 2019;9(6): DOI:10.3390/bs9060059. PMC6616388 31195634

[2] Michels N, Man, T., Vinck, B., et al. Dietary changes and its psychosocial moderators during the university examination period. Eur J Nutr. 2020;59(1):273–11. 30684033 10.1007/s00394-019-01906-9

[3] Abebe AM, Kebede YG, Mengistu F. Prevalence of stress and associated factors among regular students at Debre Birhan governmental and nongovernmental health science colleges North Showa Zone, Amhara Region, Ethiopia 2016. Psychiatry J. 2018;7534937. 30246015 10.1155/2018/7534937 PMC6139197

[4] Stallman HM, Hurst CP. The university stress scale: measuring domains and extent of stress in university. Students. Australian Psychologist. 2016;51(2): DOI:10.1111/ap.12127

[5] Reddy KJ, Menon KR, Thttil A. Academic stress and its sources among university students. Pharmacol J. 2018;11(1).

[6] Abdulghani HM, AlKanhal, A. A., Mahmoud, E. S., et al. Stress and its effects on medical students: a cross-sectional study at a college of medicine in Saudi Arabia. J Health Popul Nutr. 2011;29(5):516–522. 22106758 10.3329/jhpn.v29i5.8906 PMC3225114

[7] Singh G, Hankins M, Weinman JA. Does medical school cause health anxiety and worry in medical students? Med Educ. 2004;38(5):479–481. 15107081 10.1046/j.1365-2929.2004.01813.x

[8] Stewart SM, Lam, T.H., Betson, C.L., et al. A prospective analysis of stress and academic performance in the first two years of medical school. Med Educ. 1999;33(4):243–250. 10336754 10.1046/j.1365-2923.1999.00294.x

[9] Quynh HHN, Tanasugarn, C., Kengganpanich, M, et al. Mental well-being, and coping strategies during stress for preclinical medical students in Vietnam. J Popul Social Stud [JPSS]. 2020;28(2):116–129.

[10] Hill MR, Goicochea S, Merlo LJ. In their own words: stressors facing medical students in the millennial generation. Med Educ Online. 2018;23(1):1530558. 30286698 10.1080/10872981.2018.1530558 PMC6179084

[11] Beharu WT. Psyhological factors affecting students academic performance among freshman psychology students in dire dawa university. J Educ Pract. 2018;9(4):59–65.

[12] Uka A. Student satisfaction as an indicator of quality in higher education. J Educ Instructional Stud World. 2014;4(3 6–10).

[13] Odriozola-González P, Planchuelo-Gómez Á, Irurtia MJ, et al. Psychological effects of the COVID-19 outbreak and lockdown among students and workers of a Spanish university. Psychiatry Res. 2020;290:113108. 32450409 10.1016/j.psychres.2020.113108 PMC7236679

[14] Pulido‐Martos M, Augusto‐Landa JM, Lopez‐Zafra E. Sources of stress in nursing students: a systematic review of quantitative studies. Int Nurs Rev. 2012;59(1):15–25.

[15] Bataineh MZ. Academic stress among undergraduate students: the case of education faculty at King Saud University. Int Interdisciplinary J Educ. 2013;1(1033):1–7.

[16] White MA, Venkataraman, A., and Roehrig, A., et al. Evaluation of a behavioral self-care intervention administered through a massive open online course. Am J Health Educ. 2021 52 4 ;1–8.

[17] Fares J, Al Tabosh, H., Saadeddin, Z., et al. Stress, burnout and coping strategies in preclinical medical students. N Am J Med Sci. 2016;8(2):75. 27042604 10.4103/1947-2714.177299 PMC4791902

[18] Pham T, Bui, L., Nguyen, A, et al. The prevalence of depression and associated risk factors among medical students: an untold story in Vietnam. PloS One. 2019;14(8):e0221432. 31430339 10.1371/journal.pone.0221432 PMC6701769

[19] Do QD, Tasanapradit P. Depression and stress among the first-year medical students in the university of medicine and pharmacy, Hochiminh city, Vietnam. J Health Res. 2008;22:1–4.

[20] Robinaugh DJ, Hoekstra, R.H., Toner, E.R., et al. The network approach to psychopathology: a review of the literature 2008-2018 and an agenda for future research. Psychol Med. 2020;50(3):353–366. 31875792 10.1017/S0033291719003404 PMC7334828

[21] Smith KE, Crosby, R.D., Wonderlich, S.A., et al. Network analysis: an innovative framework for understanding eating disorder psychopathology. Int J Eat Disord. 2018;51(3):214–222. 29451959 10.1002/eat.22836 PMC5946321

[22] Dalege J, Borsboom,D., van Harreveld,F., et al. Network analysis on attitudes: a brief tutorial. Soc Psychol Personal Sci. 2017;8(5):528–537. 28919944 10.1177/1948550617709827 PMC5582642

[23] Epskamp S, Borsboom D, Fried EI. Estimating psychological networks and their accuracy: a tutorial paper. Behav Res Methods. 2018;50(1):195–212. 28342071 10.3758/s13428-017-0862-1 PMC5809547

[24] Fan A.P., Tran, D.T., and Kosik, R.O., et al. Medical education in Vietnam. Med Teach. 2012;34(2):103–107. 22288987 10.3109/0142159X.2011.613499

[25] Constantin MA, and Cramer AAOJ. Sample size recommendations for estimating cross-sectional network models (Tilburg University). 2020.

[26] Hoang T.M.N., and Nguyen T.K. Factor analysis of psychological factors of study motivation among students of of college of economics at can tho university (Vietnamese languague). J Sci Can Tho Univ. 2016 46 ;107–115 doi:10.22144/ctu.jvn.2016.575.

[27] Stipek DJ. *Motivation to learn: from theory to practice*. 1993 (ERIC) 1993 292.

[28] Preedy V.R., and Watson R.R. Likert Scale. Handbook of disease burdens and quality of life measures. New York: Springer; 2010 4248–4248 doi:10.1007/978-0-387-78665-0_6017.

[29] Gao W, Ping S, Liu X. Gender differences in depression, anxiety, and stress among college students: a longitudinal study from China. J Affect Disord. 2020;263:292–300. 31818792 10.1016/j.jad.2019.11.121

[30] Friedman J, Hastie T, Tibshirani R. Package ‘glasso’: graphical lasso: estimation of Gaussian graphical models. 2019 [cited 2020 Dec 12]. Available from: https://cran.r-project.org/web/packages/glasso/glasso.pdf

[31] Beard C, Millner, A.J., Forgeard, M.J., et al. Network analysis of depression and anxiety symptom relationships in a psychiatric sample. Psychol Med. 2016;46(16):3359–3369. 27623748 10.1017/S0033291716002300 PMC5430082

[32] Friedman J, Hastie T, Tibshirani R. Package ‘bootnet’: bootstrap methods for various network estimation routines. 2019 [cited 2021 Jul 4]. Available from: https://cran.r-project.org/web/packages/bootnet/bootnet.pdf

[33] Almaiah MA, Alyoussef IY. Analysis of the effect of course design, course content support, course assessment and instructor characteristics on the actual use of E-learning system. Ieee Access. 2019;7:171907–171922.

[34] Street SE, Gilliland KO, McNeil C, et al. The flipped classroom improved medical student performance and satisfaction in a pre-clinical physiology course. Med Sci Educator. 2015;25(1):35–43.

[35] Gurpinar E, Gilliland KO, McNeil C, et al. Do learning approaches of medical students affect their satisfaction with problem-based learning?. Adv Physiol Educ. 2013;37(1):85–88. 23471254 10.1152/advan.00119.2012

[36] Weerasinghe I.M.S, and Fernando R.L.S. University facilities and student satisfaction in Sri Lanka. International Journal of Educational Management. 2018 32 5 doi:10.1108/IJEM-07-2017-0174.

[37] Tessema MT, Ready K, Yu W. Factors affecting college students’ satisfaction with major curriculum: evidence from nine years of data. Int J Humanities Social Sci. 2012;2(2):34–44.

[38] Backović D.V., Živojinović, J.I., and Maksimović, J., et al. Gender differences in academic stress and burnout among medical students in final years of education. Psychiatr Danub. 2012;24(2):175–181. 22706416

[39] Calvarese M. The effect of gender on stress factors: an exploratory study among university students. Soc Sci. 2015;4(4):1177–1184.

[40] Tran Q.A., Dunne, M.P., and Vo, T.V. *et al*, et al. Adverse childhood experiences and the health of university students in eight provinces of Vietnam. Asia Pac J Public Health. 2015;27(8_suppl):26S–32S. 26047629 10.1177/1010539515589812

[41] Tran Q.A. Factors associated with mental health of medical students in Vietnam: A national study PhD thesis. Queensland University of Technology; 2015.

[42] Safhi M.A., Alafif, R.A., and Alamoudi, N.M., et al. The association of stress with sleep quality among medical students at King Abdulaziz University. J Family Med Prim Care. 2020;9(3):1662–1667. 32509668 10.4103/jfmpc.jfmpc_745_19 PMC7266176

[43] Choi J. Impact of stress levels on eating behaviors among college students. Nutrients. 2020;12(5): DOI:10.3390/nu12051241 PMC7284653 32349338

[44] Alotaibi A.D., Alosaimi, F.M., and Alajlan, A.A. *et al*. The relationship between sleep quality, stress, and academic performance among medical students. J Family Community Med. 2020;27(1):23–28. 32030075 10.4103/jfcm.JFCM_132_19 PMC6984036

[45] Briguglio M, Vitale JA, Galentino R, et al. Healthy eating, physical activity, and sleep hygiene (HEPAS) as the winning triad for sustaining physical and mental health in patients at risk for or with neuropsychiatric disorders: considerations for clinical practice. Neuropsychiatr Dis Treat. 2020;16:55–70. 32021199 10.2147/NDT.S229206 PMC6955623

[46] Pisaniello MS, Asahina, A.T., Bacchi, S., et al. Effect of medical student debt on mental health, academic performance and specialty choice: a systematic review. BMJ Open. 2019;9(7):e029980. 10.1136/bmjopen-2019-029980 PMC6609129 31270123

[47] Schultchen D, Reichenberger, J., Mittl, T., et al. Bidirectional relationship of stress and affect with physical activity and healthy eating. Br J Health Psychol. 2019;24(2):315–333. 30672069 10.1111/bjhp.12355 PMC6767465

[48] Garg K, Agarwal M, Dalal PK. Stress among medical students: a cross-sectional study from a North Indian medical university. Indian J Psychiatry. 2017;59(4):502–504. 29497197 10.4103/psychiatry.IndianJPsychiatry_239_17 PMC5806334

[49] Waqas A, Khan S, Sharif W, et al. Association of academic stress with sleeping difficulties in medical students of a Pakistani medical school: a cross-sectional survey. PeerJ. 2015;3:e840. 25802809 10.7717/peerj.840 PMC4369327

[50] Hoang T, Lee J, Kim J. Differences in Dietary Patterns Identified by the Gaussian Graphical Model in Korean Adults With and Without a Self-Reported Cancer Diagnosis. J Acad Nutr Diet. 2021;1484–1496.e3. DOI:10.1016/j.jand.2020.11.006 33288494

[51] Gunathilake M., Lee, J., and Choi, I.J., et al. Identification of dietary pattern networks associated with gastric cancer using gaussian graphical models: a case-control study. Cancers (Basel). 2020;12(4 1044). DOI:10.3390/cancers12041044. PMC7226381 32340406

[52] Iqbal K., Buijsse, B., and Wirth, J., et al. Gaussian graphical models identify networks of dietary intake in a German adult population. J Nutr. 2016;146(3):646–652. 26817715 10.3945/jn.115.221135

