# Supplementary figures and images for: Network analysis of psychological factors related to academic pressure faced by medical students in the central and highland regions of Vietnam
Source: Med Educ Online. 2021 Nov 25;27(1):2007577. doi: 10.1080/10872981.2021.2007577 (PMC9639564; doi:10.1080/10872981.2021.2007577)

Figure S1
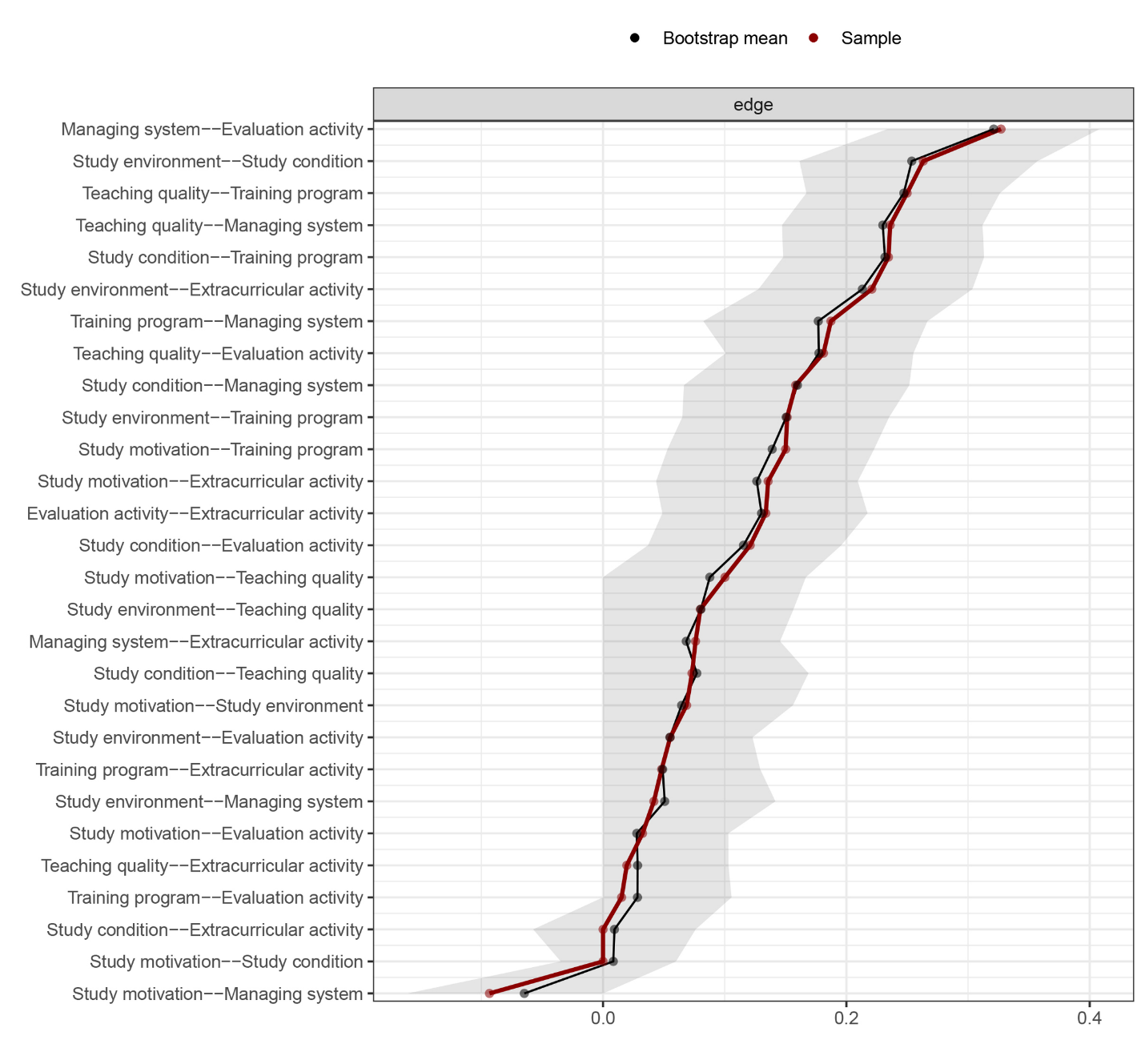

Supplement: Supplemental Material [file ZMEO_A_2007577_SM4628.zip › Supplementary files/Figure_S1.docx]

Figure S2A
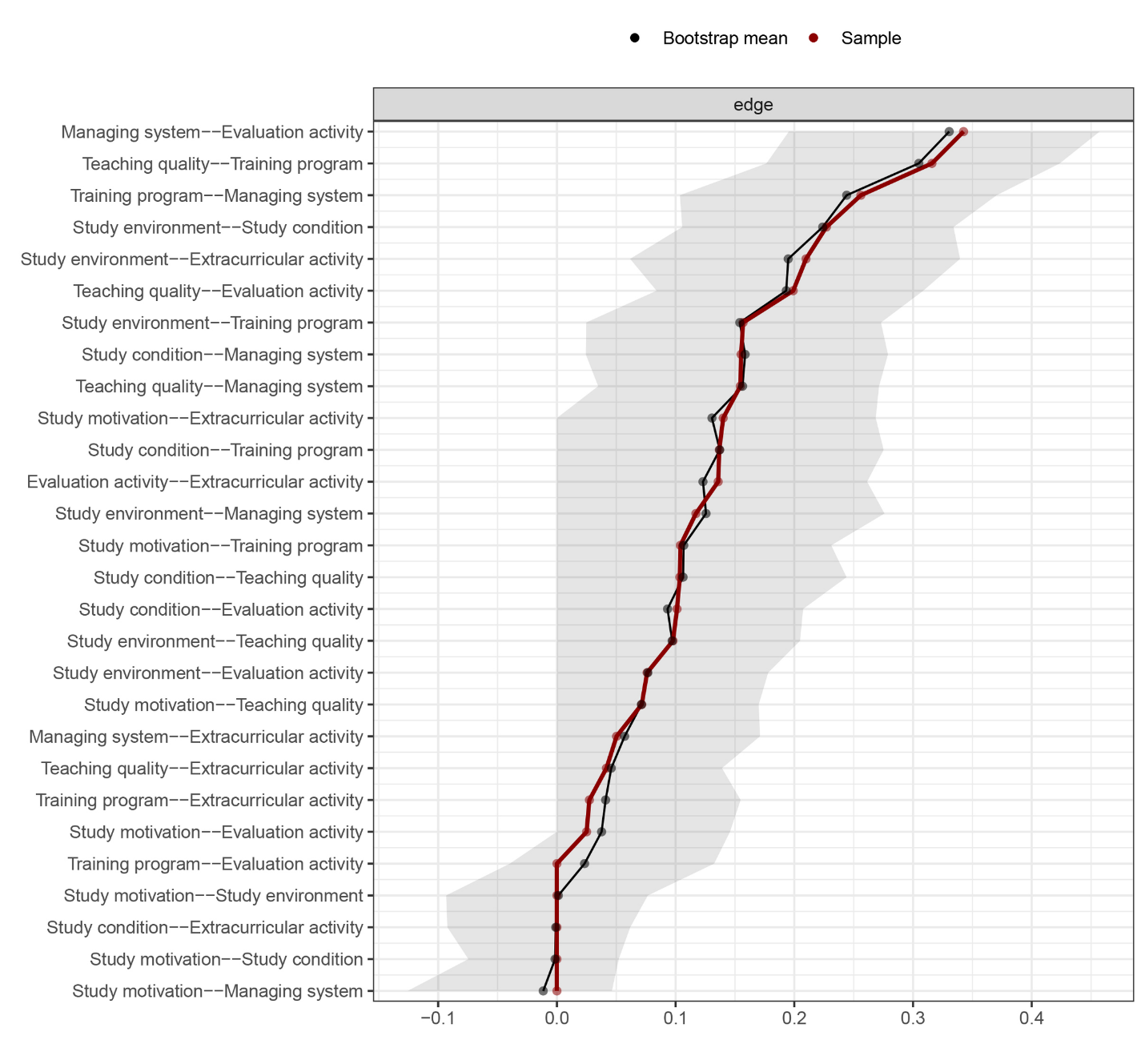

Supplement: Supplemental Material [file ZMEO_A_2007577_SM4628.zip › Supplementary files/Figure_S2A.docx]

Figure S2B
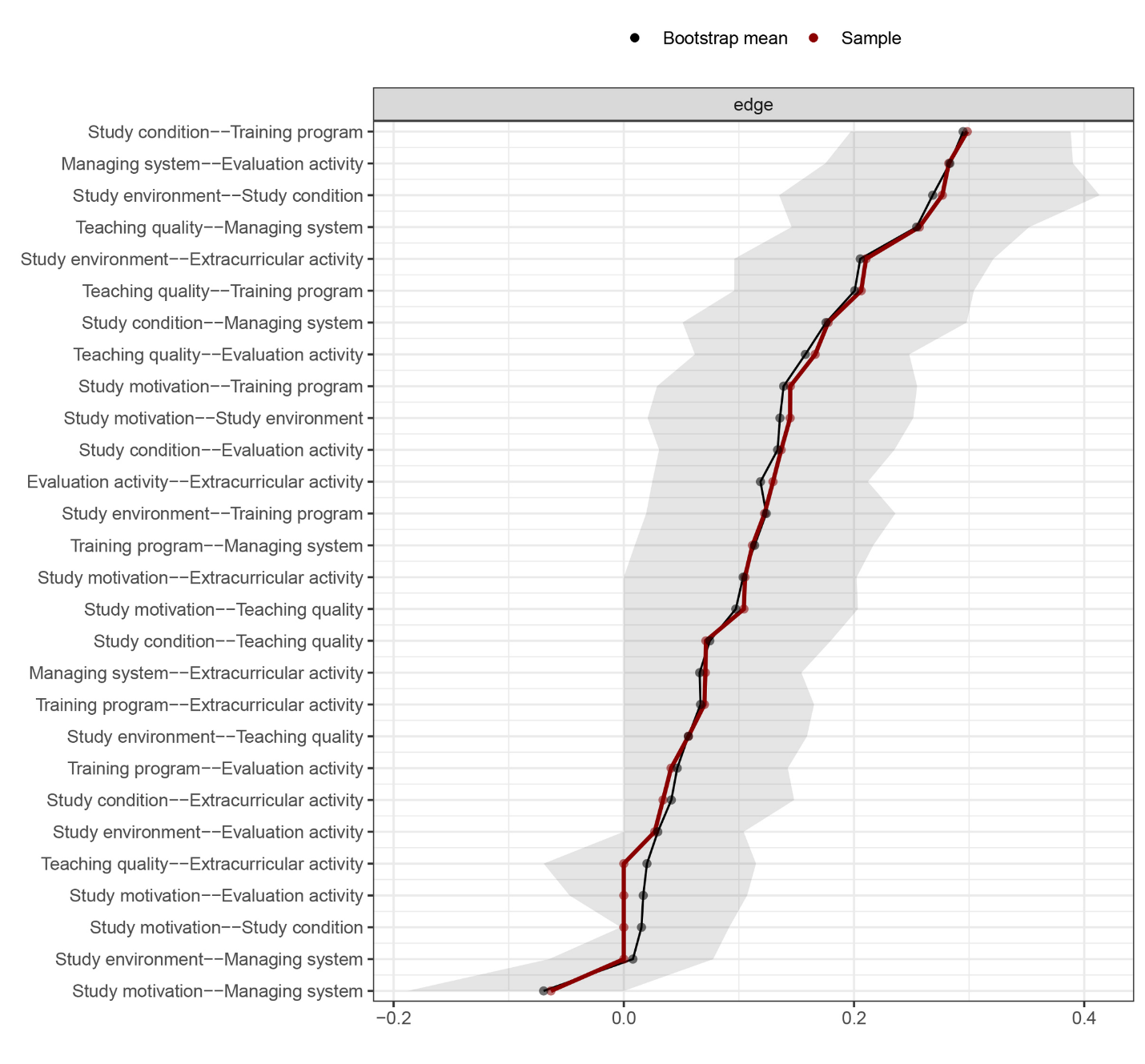

Supplement: Supplemental Material [file ZMEO_A_2007577_SM4628.zip › Supplementary files/Figure_S2B.docx]

Figure S3A
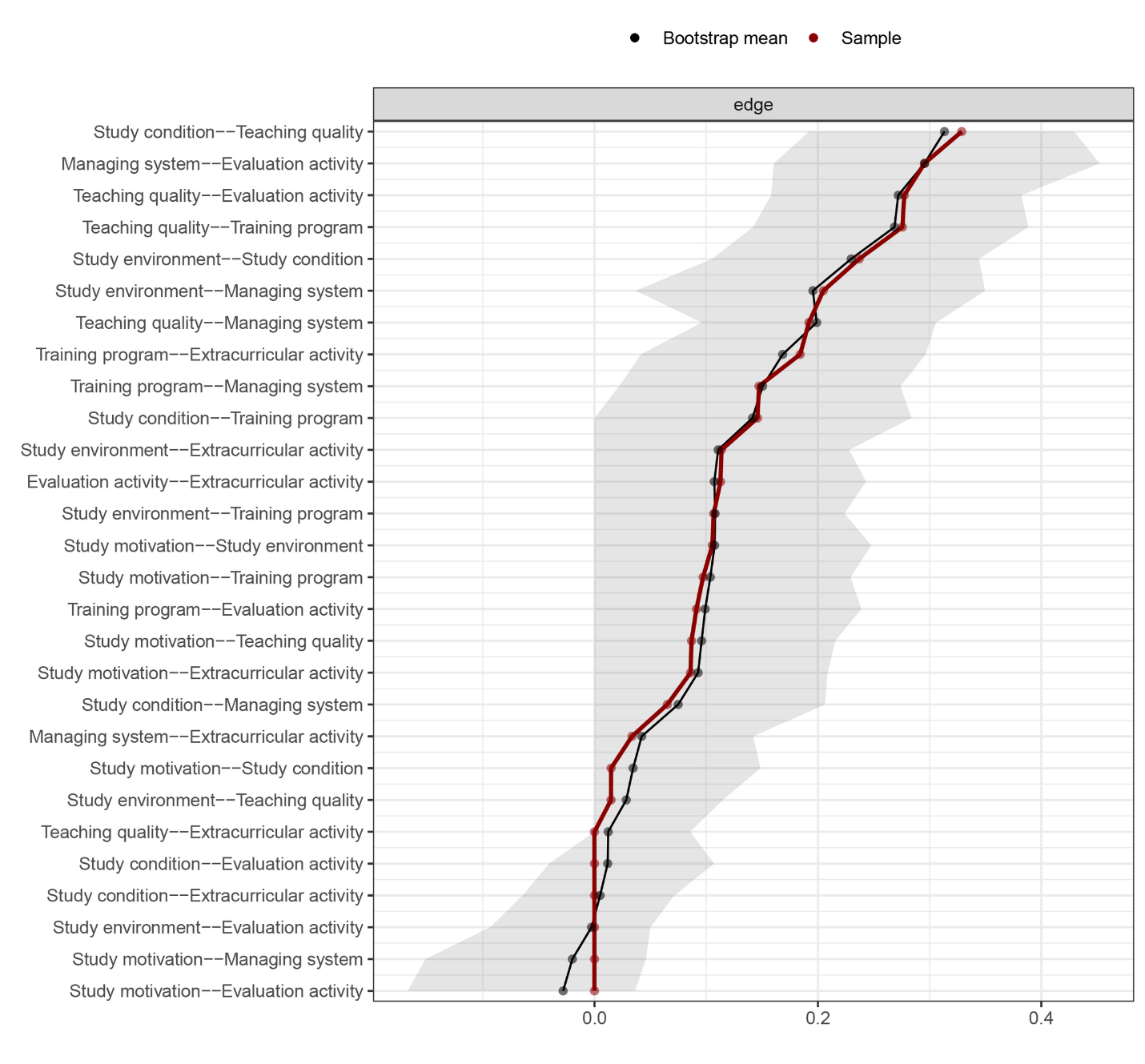

Supplement: Supplemental Material [file ZMEO_A_2007577_SM4628.zip › Supplementary files/Figure_S3A.docx]

Figure S3B
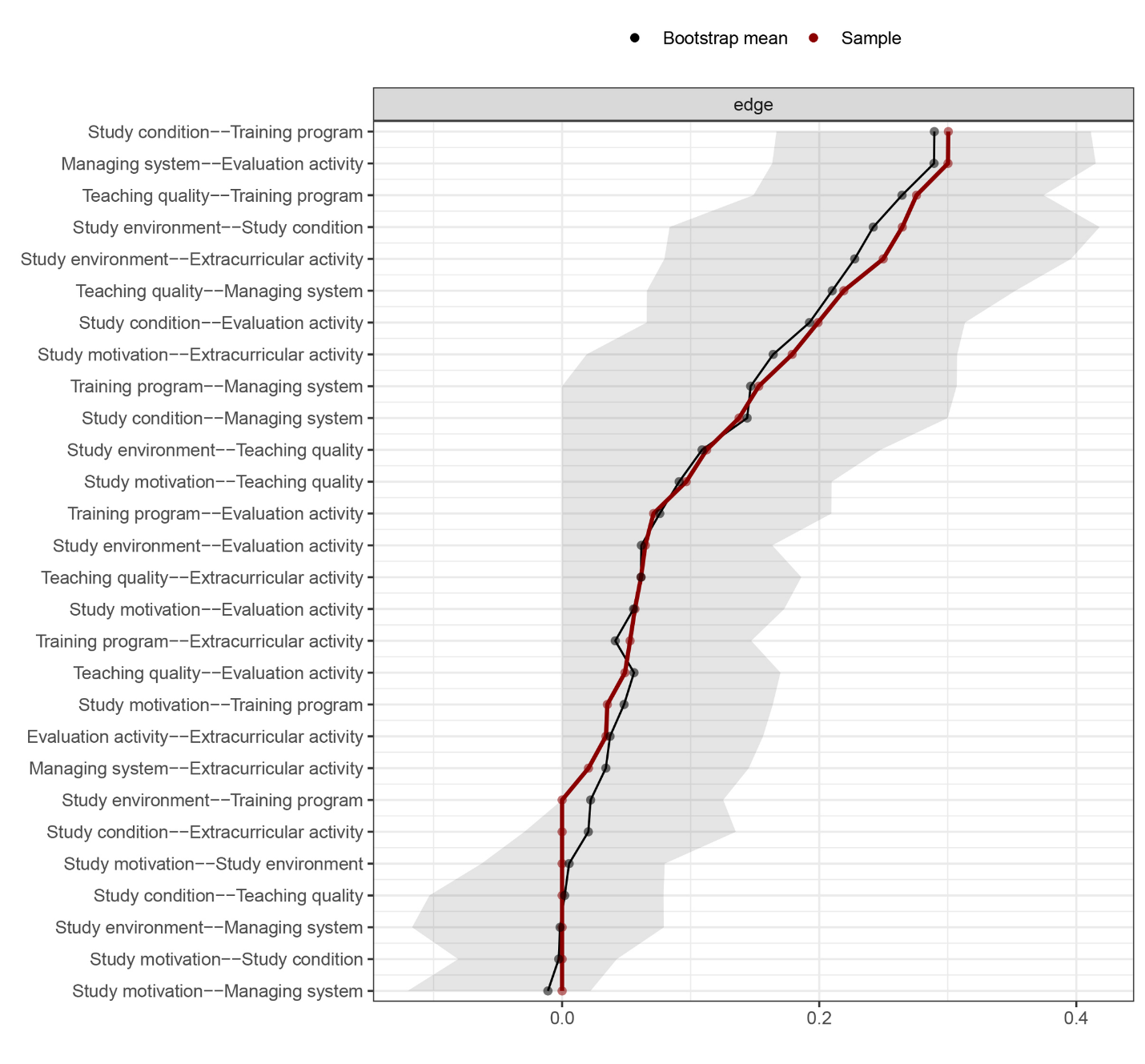

Supplement: Supplemental Material [file ZMEO_A_2007577_SM4628.zip › Supplementary files/Figure_S3B.docx]

Figure S3C
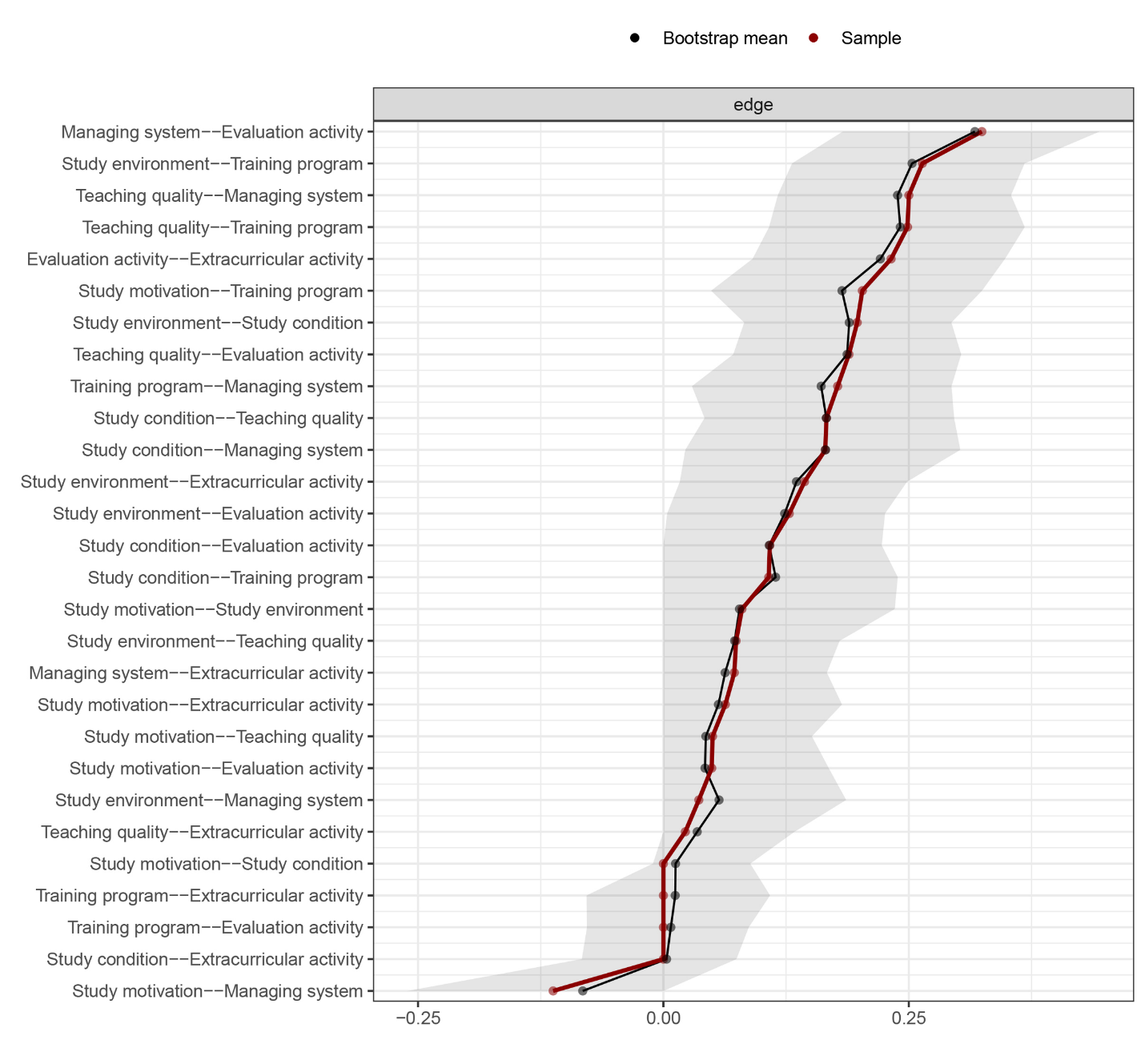

Supplement: Supplemental Material [file ZMEO_A_2007577_SM4628.zip › Supplementary files/Figure_S3C.docx]

Figure S4
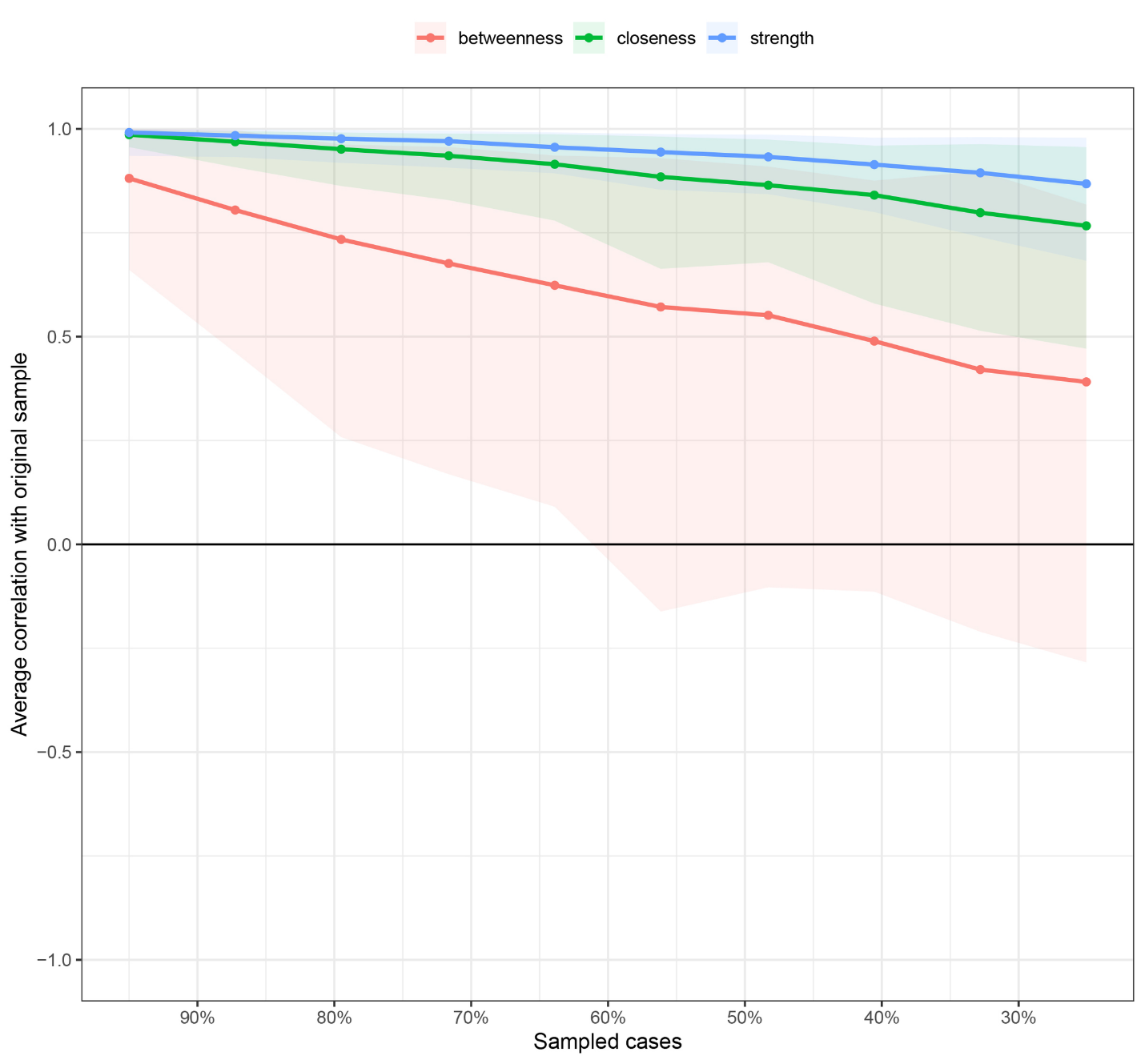

Supplement: Supplemental Material [file ZMEO_A_2007577_SM4628.zip › Supplementary files/Figure_S4.docx]

Figure S5A
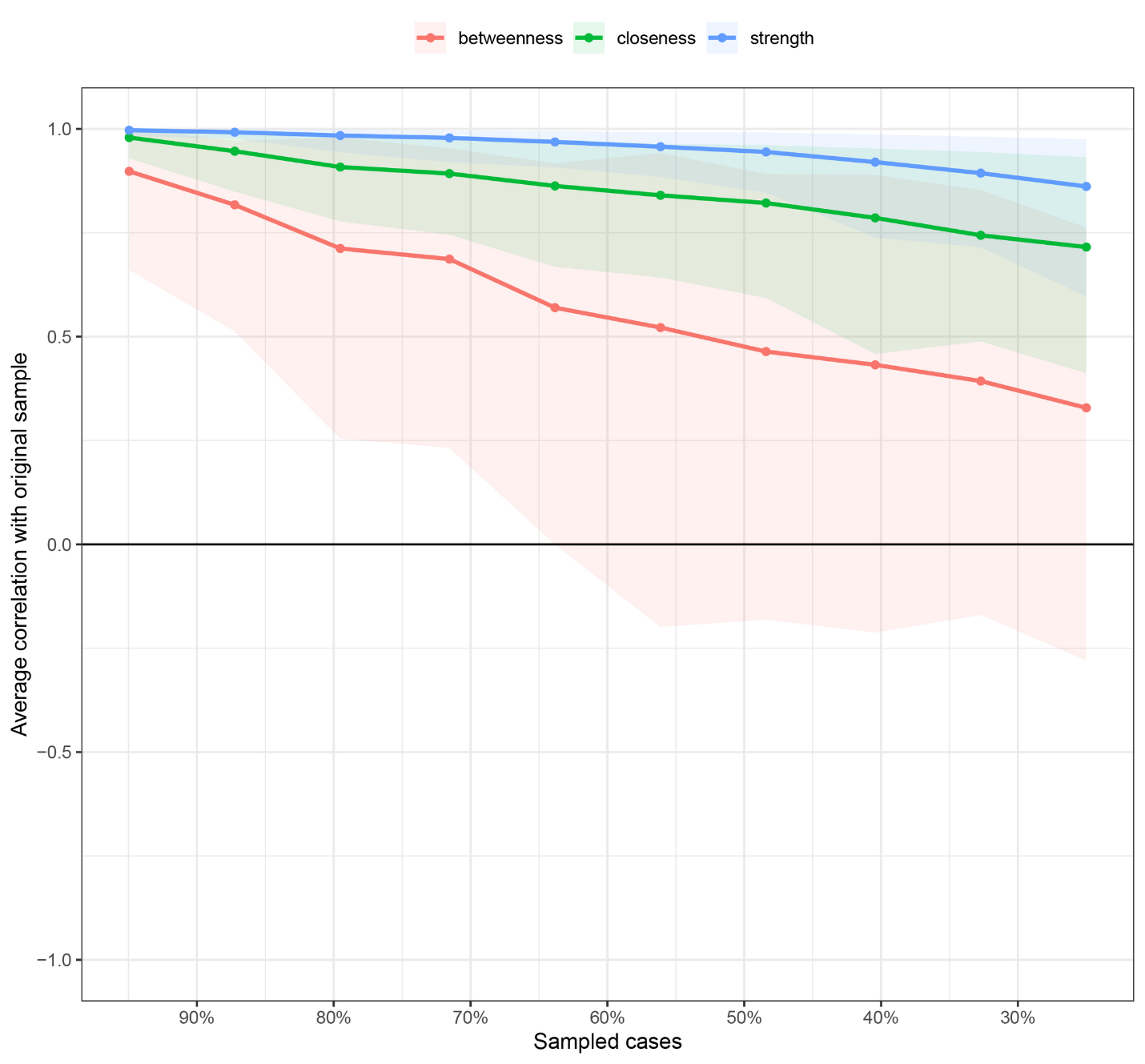

Supplement: Supplemental Material [file ZMEO_A_2007577_SM4628.zip › Supplementary files/Figure_S5A.docx]

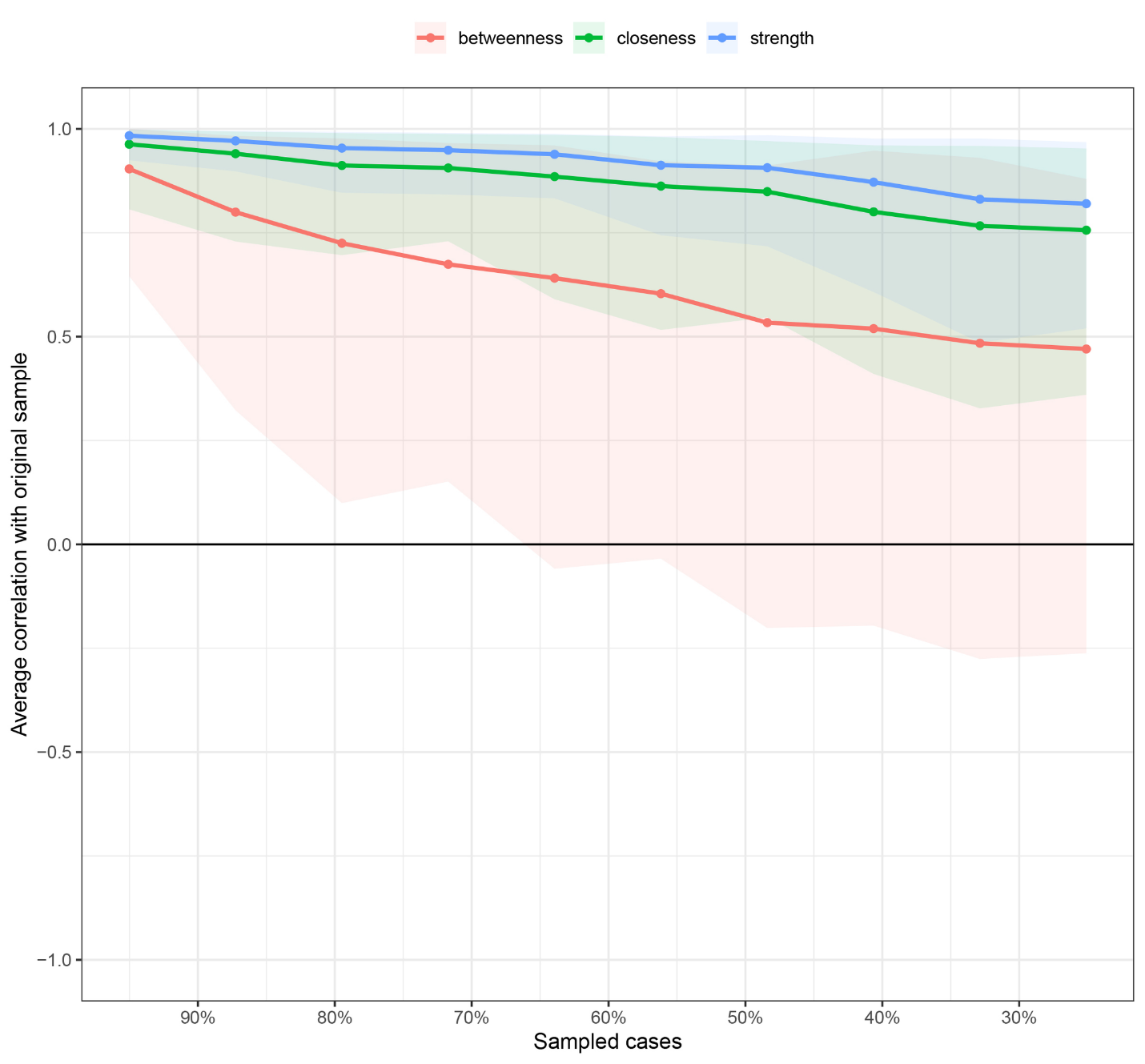

Supplement: Supplemental Material [file ZMEO_A_2007577_SM4628.zip › Supplementary files/Figure_S5B.docx]

Figure S6A
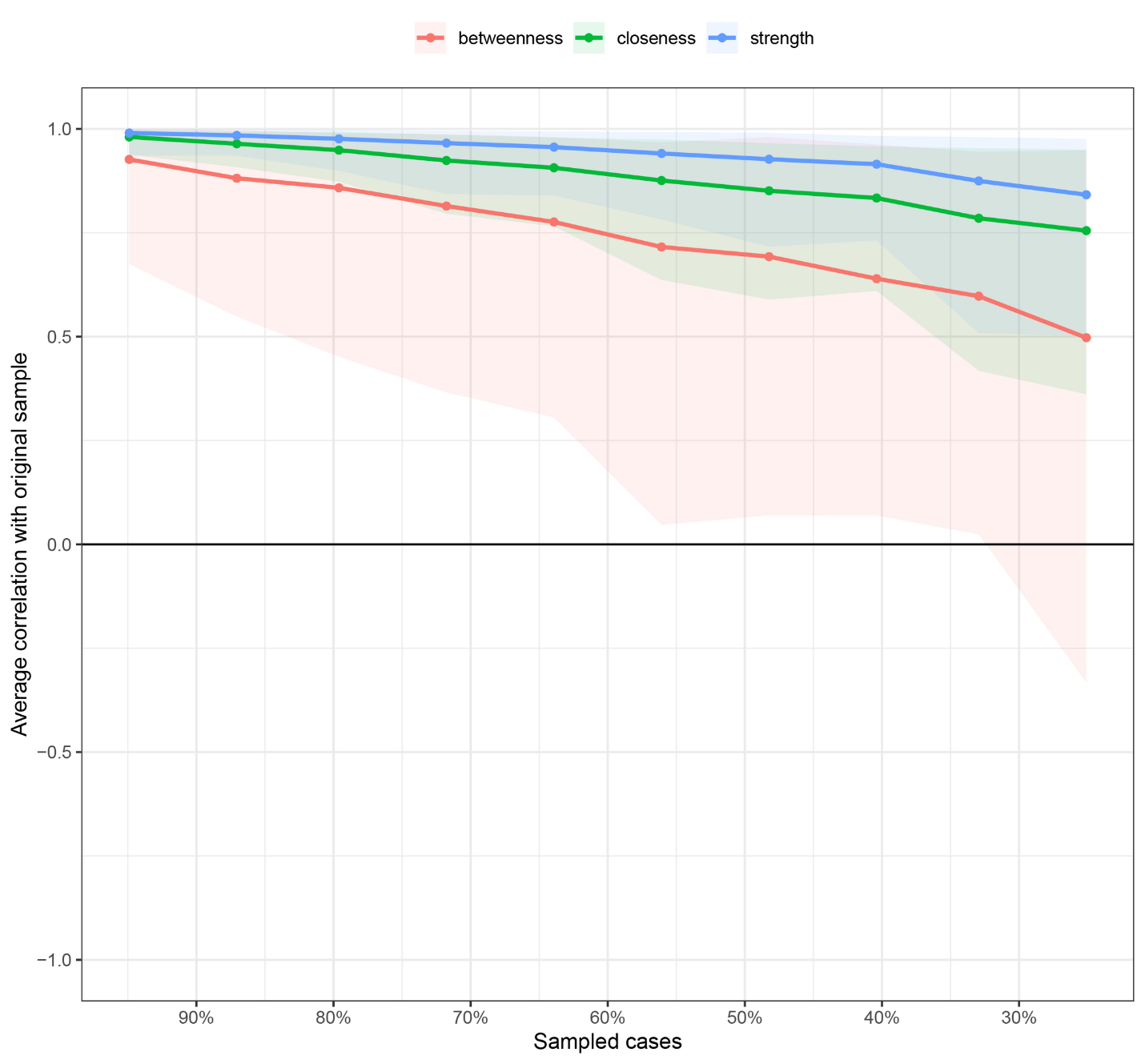

Supplement: Supplemental Material [file ZMEO_A_2007577_SM4628.zip › Supplementary files/Figure_S6A.docx]

Figure S6B
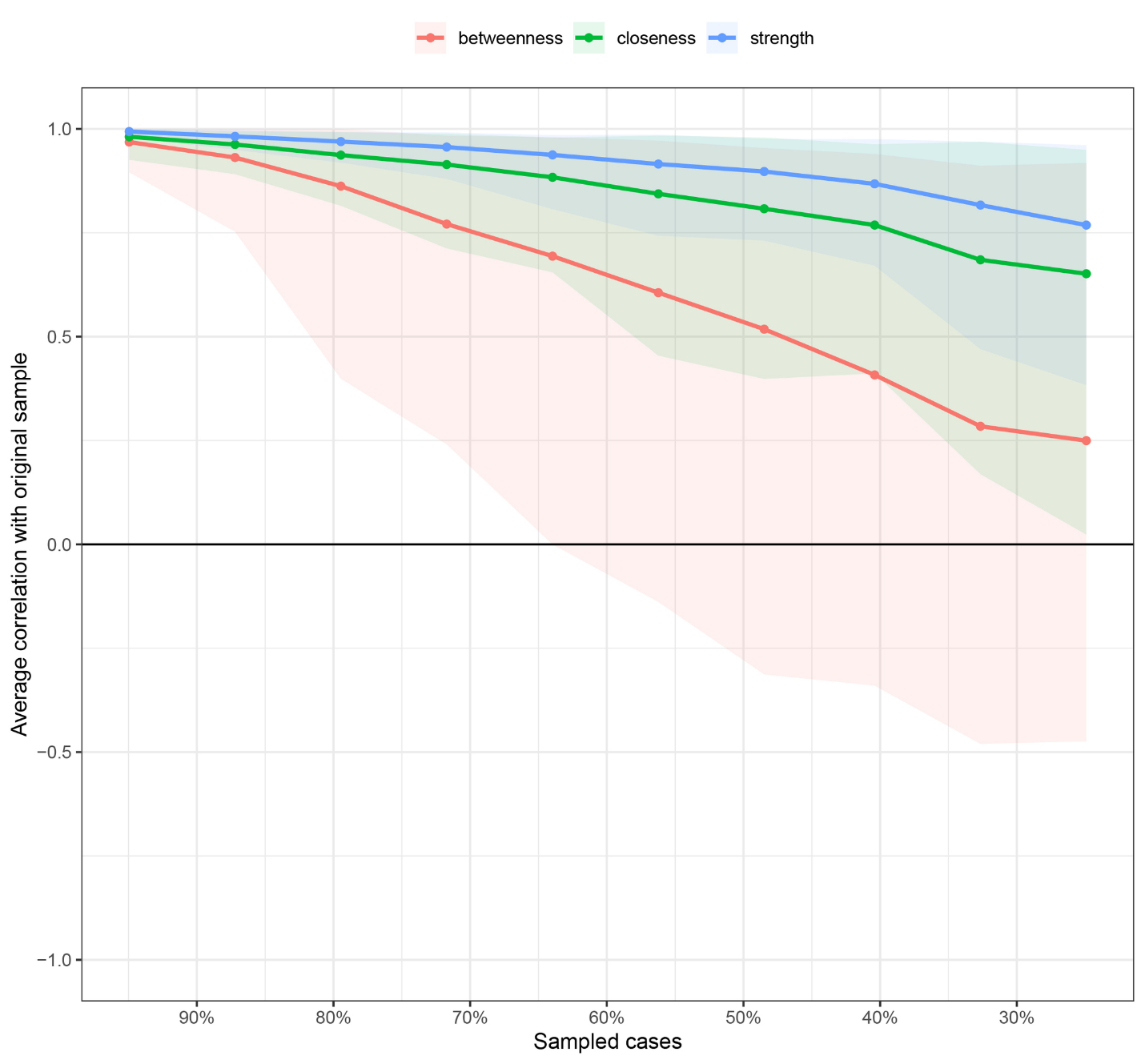

Supplement: Supplemental Material [file ZMEO_A_2007577_SM4628.zip › Supplementary files/Figure_S6B.docx]

Figure S6C
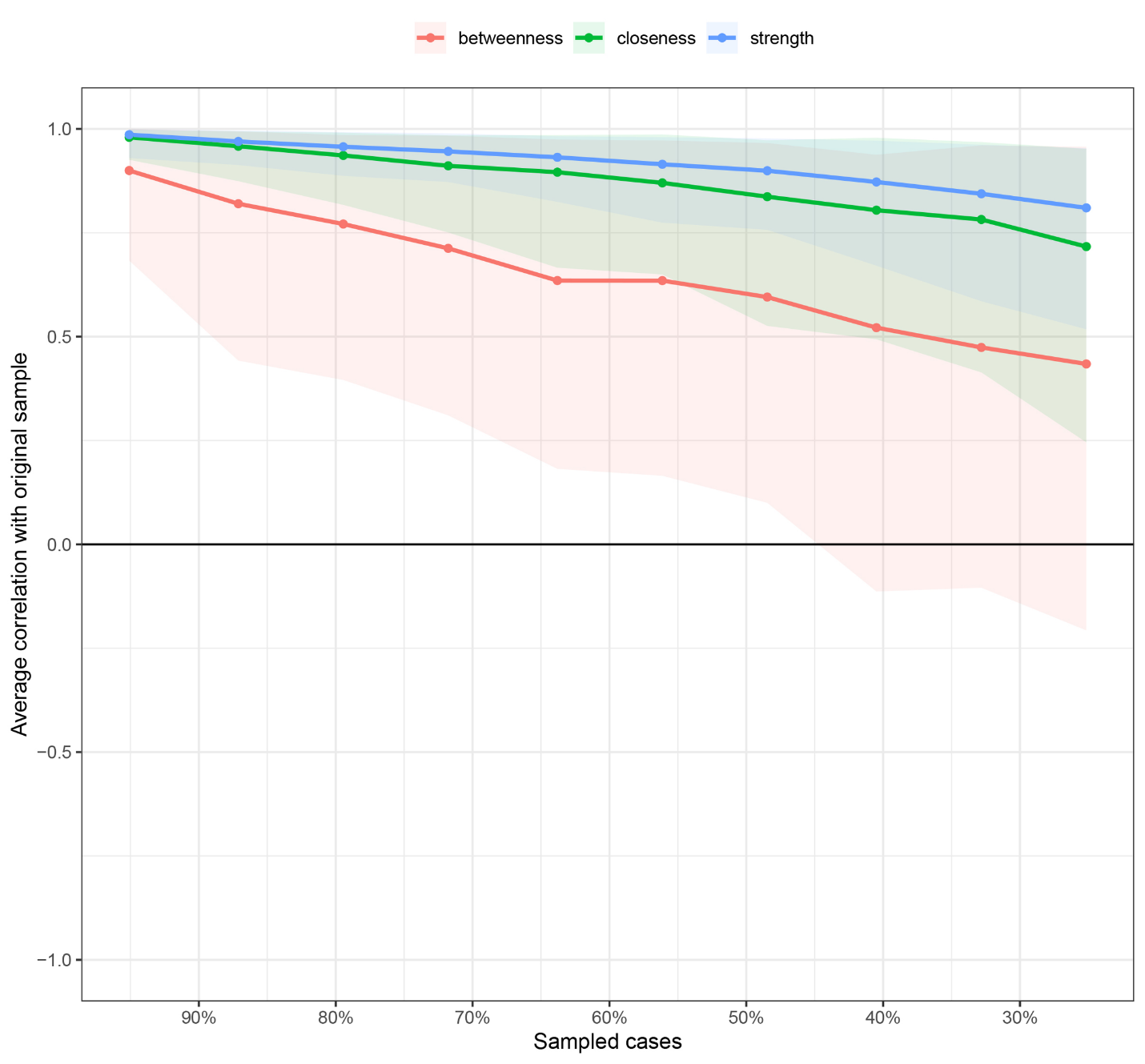

Supplement: Supplemental Material [file ZMEO_A_2007577_SM4628.zip › Supplementary files/Figure_S6C.docx]
